# The UBC9 E2 SUMO Conjugating Enzyme Binds the PR-Set7 Histone Methyltransferase to Facilitate Target Gene Repression

**DOI:** 10.1371/journal.pone.0022785

**Published:** 2011-07-29

**Authors:** Tanya M. Spektor, Lauren M. Congdon, Chendhore S. Veerappan, Judd C. Rice

**Affiliations:** Department of Biochemistry and Molecular Biology, University of Southern California Keck School of Medicine, Los Angeles, California, United States of America; Ludwig-Maximilians-Universität München, Germany

## Abstract

PR-Set7/Set8/KMT5a is a chromatin-modifying enzyme that specifically monomethylates lysine 20 of histone H4 (H4K20me1). In this study we attempted to identify PR-Set7-interacting proteins reasoning that these proteins would provide important insights into the role of PR-Set7 in transcriptional regulation. Using an unbiased yeast two-hybrid approach, we discovered that PR-Set7 interacts with the UBC9 E2 SUMO conjugating enzyme. This interaction was confirmed in human cells and we demonstrated that PR-Set7 was preferentially modified with SUMO1 *in vivo*. Further *in vitro* studies revealed that UBC9 directly binds PR-Set7 proximal to the catalytic SET domain. Two putative SUMO consensus sites were identified in this region and both were capable of being SUMOylated *in vitro*. The absence of either or both SUMO sites did not perturb nuclear localization of PR-Set7. By employing whole genome expression arrays, we identified a panel of genes whose expression was significantly altered in the absence of PR-Set7. The vast majority of these genes displayed increased expression strongly suggesting that PR-Set7 predominantly functions as a transcriptional repressor. Importantly, the reduction of UBC9 resulted in the consistent derepression of several of these newly identified genes regulated by PR-Set7. Our findings indicate that direct interaction with UBC9 facilitates the repressive effects of PR-Set7 at specific target genes, most likely by SUMOylating PR-Set7.

## Introduction

The eukaryotic genome is organized and packaged in the nucleus into a structure known as chromatin, composed of DNA and DNA-associated proteins. The nucleosome is the fundamental repeating subunit of chromatin consisting of 146 bp of DNA wrapped around an octamer of the canonical histone proteins H2A, H2B, H3 and H4. The N-terminal tails of the histones protrude from the nucleosome to interact with the nuclear environment [1]. Certain amino acids within these histone tails are targets for various post-translational modifications, such as acetylation, phosphorylation and methylation, that are created by distinct chromatin-modifying enzymes [2]. Increasing evidence indicates that specific histone modifications play important roles in DNA-templated processes including transcriptional regulation, DNA repair, replication and recombination.

PR-Set7/Set8/KMT5a is a histone modifying enzyme that specifically and selectively monomethylates lysine 20 of histone H4 (H4K20me1) [3], [4], [5], [6]. Previous reports demonstrated that PR-Set7 and H4K20me1 are essential as depletion of PR-Set7 results in aberrant chromosomal abnormalities and embryonic lethality [7], [8], [9], [10]. These phenotypes are likely caused, in part, by cell cycle defects as several recent findings indicate that PR-Set7 has important roles in mammalian cell cycle progression [11], [12], [13], [14], [15]. In addition to the cell cycle, PR-Set7 and H4K20me1 are also involved in the transcriptional regulation of specific genes. Both were originally postulated to function in transcriptionally repressive pathways as immunofluorescence microscopy studies demonstrated that they are typically excluded from actively transcribed chromatin [5], [16]. However, subsequent genome-wide and gene-specific studies demonstrated that the H4K20me1 modification was enriched in the 5′ end of many actively transcribed genes suggesting a role for PR-Set7 in activation pathways [17], [18], [19]. Due to these apparently conflicting reports, it currently remains unclear how PR-Set7 functions in the regulation of specific genes. Since chromatin-modifying enzymes are usually components of large multi-protein complexes, we reasoned that the identification of PR-Set7-interacting proteins would lead to important insights into the function of PR-Set7 in transcriptional regulation.

In this report, we employed an unbiased yeast two-hybrid approach to discover that PR-Set7 directly binds the UBC9 E2 SUMO conjugating enzyme. UBC9 functions to covalently modify specific proteins with a 100 amino acid small ubiquitin-related modifier (SUMO) peptide (reviewed in [20]). There are four different SUMO forms (1–4) and, once conjugated to the target protein, are capable of creating multi-peptide chains. A diverse array of substrate proteins are known to be SUMOylated *in vivo* and, in many cases, the covalent addition of SUMO can dramatically alter the localization and function of the modified protein. Although these substrate proteins are involved in different biological pathways, they usually share a conserved SUMO consensus motif, ΨKxE/D (where Ψ is bulky hydrophobic residue, K is the target of SUMOylation and x is any amino acid), that is directly recognized by UBC9. We identified two putative SUMO consensus motifs within PR-Set7 and demonstrated that each can be SUMOylated *in vitro*. Although the absence of these SUMO sites did not affect the nuclear localization of PR-Set7, the reduction of UBC9 resulted in the derepression of several newly identified genes regulated by PR-Set7. Collectively these findings indicate that UBC9 is necessary to facilitate the full repressive effects of PR-Set7, most likely by directly SUMOylating PR-Set7.

## Results

### Identification of UBC9 as a PR-Set7-interacting protein by yeast two-hybrid

**Figure 1:**
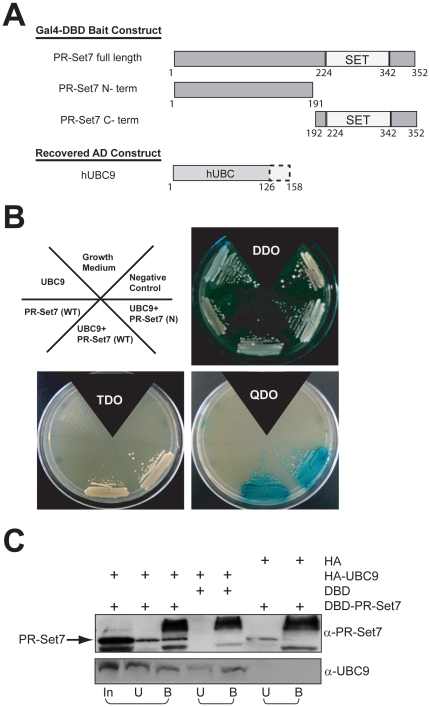
Yeast two-hybrid screen identifies UBC9 as a PR-Set7-interacting protein. (A) Schematic representation of the different Gal4-DBD-PR-Set7 bait proteins used for the yeast two-hybrid screen and the recovered AD-UBC9. (B) The indicated plasmids were co-transformed into AH109 yeast strain and grown on medium lacking: tryptophan and leucine (DDO), and histidine (TDO) and adenine with addition of X-alpha-galactosidase (QDO). Growth on TDO and QDO indicates interaction between PR-Set7 and UBC9. (C) Western analysis of HA-immunoprecipitations from yeast co-transformants expressing the indicated plasmids using PR-Set7 and UBC9 antibodies on the input (In), unbound (U) and bound (B) fractions.

A yeast two-hybrid screen using full length human PR-Set7 was used as bait to screen a human HeLa cDNA library for putative PR-Set7-interacting proteins (Figure 1A). The number of positive yeast colonies isolated from the screen was unexpectedly limited, most likely due to the slow growth phenotype of the AH109 yeast strain expressing full length PR-Set7. The slow growth phenotype was directly due to the catalytic SET domain: a bait plasmid containing only the C-terminal_192–352_ of PR-Set7 was lethal whereas a bait plasmid containing only the N-terminal_1–191_ of PR-Set7 did not alter cell growth or viability (data not shown).

One of the positive clones isolated from the screen encoded the first 126 amino acids of the human UBC9 (UBE2I) SUMO E2 conjugating enzyme (Figure 1A). Co-expression of the recovered pGADT7-AD-UBC9 plasmid with the Gal4-DBD-PR-Set7 bait plasmid in yeast confirmed the interaction due to activation of the HIS3, ADE2 and lacZ/MEL1 reporter genes present in the indicator yeast strain. Furthermore, growth of the UBC9 and PR-Set7 co-transformants on selective medium verified the interaction as the various control co-transformants failed to survive (Figure 1B). Similar results were observed when using the PR-Set7 N-terminal_1–191_ bait plasmid indicating that that catalytic SET domain is dispensable for UBC9 interaction.

To verify UBC9 and PR-Set7 interaction in yeast, co-immunoprecipitations were performed using HA-tagged UBC9 and Gal4-DBD-tagged PR-Set7 plasmids. As shown in Figure 1C, full length Gal4-DBD-PR-Set7 co-precipitated in the HA-UBC9 bound fraction compared to the controls. Collectively, these results demonstrate that UBC9 specifically and directly interacts with PR-Set7 in yeast and that the interaction occurs on the N-terminal portion of PR-Set7.

### PR-Set7 and UBC9 transiently interact in human cells

**Figure 2:**
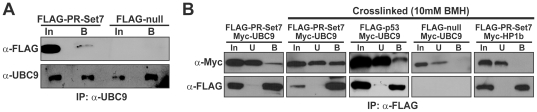
UBC9 transiently interacts with PR-Set7 in cells. (A) HEK 293 cell lysates expressing FLAG-PR-Set7 or FLAG-null were immunoprecipitated with an UBC9 antibody. Western analysis of the input (In) and bound (B) fractions were analyzed using FLAG or UBC9 antibodies. (B) HEK 293 cells co-transfected with the indicated plasmids were crosslinked using 10 µM BMH prior to FLAG-immunoprecipitations. Western analysis was performed using Myc or FLAG antibodies on the input (In), unbound (U) and bound (B) fractions.

To determine if PR-Set7 interacts with endogenous UBC9 in human cells, a FLAG-tagged full length PR-Set7 or a FLAG-null (negative control) plasmid were transfected into HEK 293 cells followed by immunoprecipitations using a UBC9 antibody. Western analysis of the material indicated that FLAG-PR-Set7, but not FLAG-null, bound endogenous UBC9 albeit weakly (Figure 2A). Consistent with this finding, recent structural and kinetics studies demonstrated that UBC9-substrate interactions, such as with p53, dissociate rapidly suggesting that UBC9 interaction with PR-Set7 is also relatively transient [21]. Therefore, we reasoned that the addition of a crosslinking reagent to the experiments would capture this potential transient interaction. To test this, HEK 293 cells were first co-transfected with a Myc-tagged UBC9 and either the FLAG-PR-Set7, FLAG-p53 (positive control) or FLAG-null (negative control) plasmids. Cells were then treated with the bismaleimidohexane (BMH) crosslinking reagent prior to FLAG-immunoprecipitations. As predicted, Western analysis demonstrated that UBC9 binding to PR-Set7 was markedly increased in the BMH treated cells compared to untreated cells (Figure 2B). Furthermore, analysis of the unbound and bound fractions suggests that UBC9 preferentially binds PR-Set7 compared to the p53 positive control. Importantly, PR-Set7 failed to bind the HP1β negative control even in the presence of BMH. Collectively, these findings indicate that PR-Set7 specifically interacts with UBC9 in human cells.

### The N-terminal portion of PR-Set7 is required for direct interaction with UBC9

**Figure 3:**
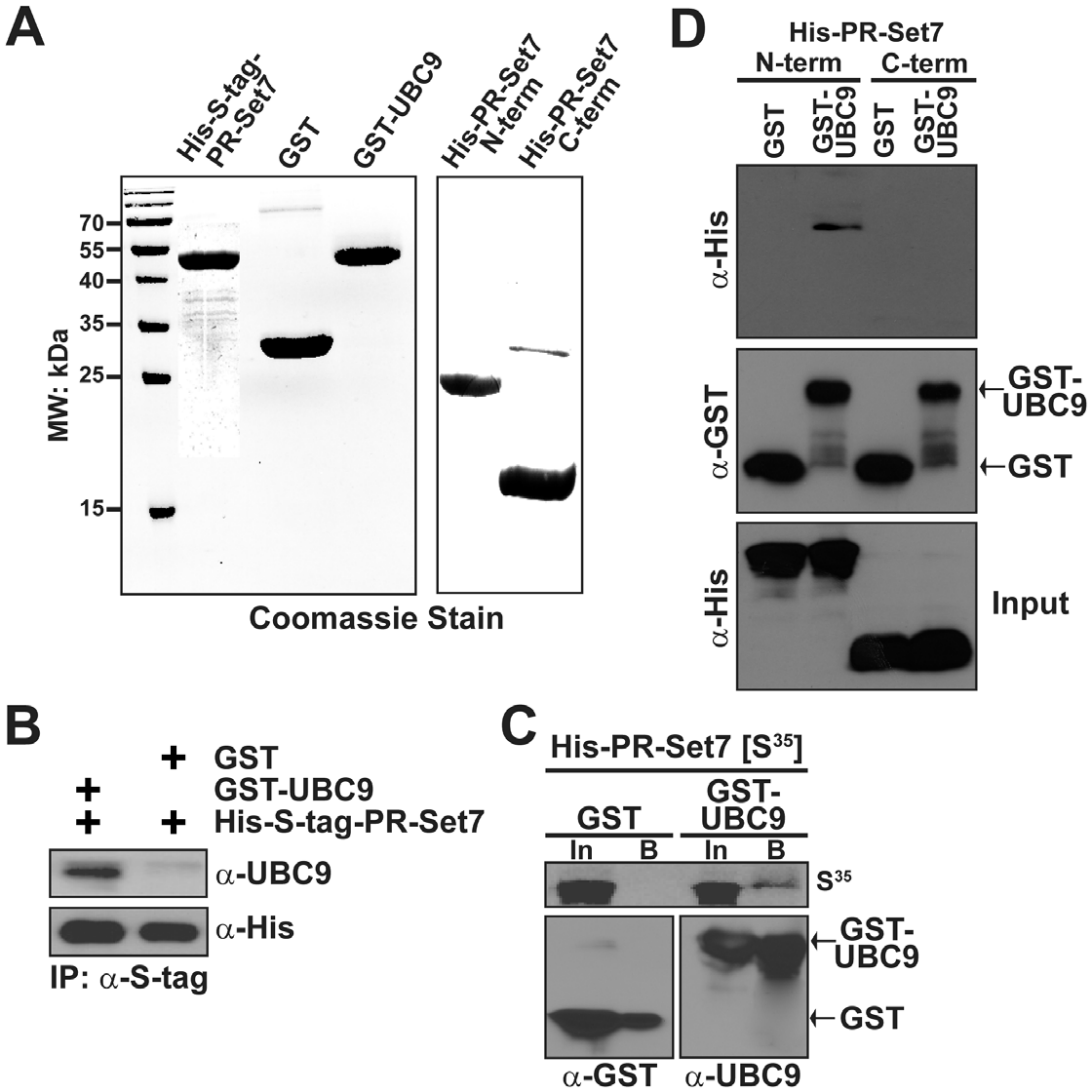
UBC9 directly binds the N-terminal region of PR-Set7. (A) The indicated recombinant fusion proteins were expressed in *E. coli*, purified by affinity chromatography and fractionated by SDS-PAGE. (B) Purified recombinant S-tag-PR-Set7 was incubated with GST-UBC9 or GST alone prior to an S-tag immunoprecipitation. Western analysis of the bound material was performed using UBC9 and His antibodies. (C) ^35^S-labeled PR-Set7 was incubated with GST-UBC9 or GST alone prior to GST pull downs. PR-Set7 binding was determined by autoradiography. Western analysis of the input (In) and bound (B) fractions were performed using GST antibodies to confirm equal loading. (D) Purified recombinant His-tagged N- and C-terminal PR-Set7 proteins were incubated with GST-UBC9 or GST alone prior to GST pull downs. Western analysis of the bound fractions was performed using His and GST antibodies.

To confirm that UBC9 and PR-Set7 directly interact, *in vitro* binding assays were performed. Full length recombinant His-S-tagged PR-Set7 and GST-tagged UBC9 proteins were expressed and purified from bacteria (Figure 3A). Following their incubation, PR-Set7 was immunoprecipitated using an S-tag antibody prior to Western analysis of the bound material. As shown in Figure 3B, recombinant PR-Set7 bound GST-UBC9 but not GST alone demonstrating a direct interaction between PR-Set7 and UBC9 *in vitro*. In a reciprocal experiment, *in vitro* translated ^35^S-labeled full length PR-Set7 was incubated with GST-UBC9 or GST alone prior to a GST pull down. Autoradiography of the SDS-PAGE fractionated input and bound material confirmed that PR-Set7 directly binds UBC9 *in vitro* (Figure 3C).

To determine the region of PR-Set7 required for binding UBC9, similar GST-UBC9 pull downs were performed following incubation with either recombinant His-tagged N-terminal_1–191_ or C-terminal_192–352_ fragments of PR-Set7 (Figure 1A). Western analysis of the bound material indicated that only the N-terminal portion of PR-Set7 is required for direct interaction with UBC9 (Figure 3D), consistent with the yeast two-hybrid results (Figure 1B).

### SUMO1-selective modification of PR-Set7

**Figure 4:**
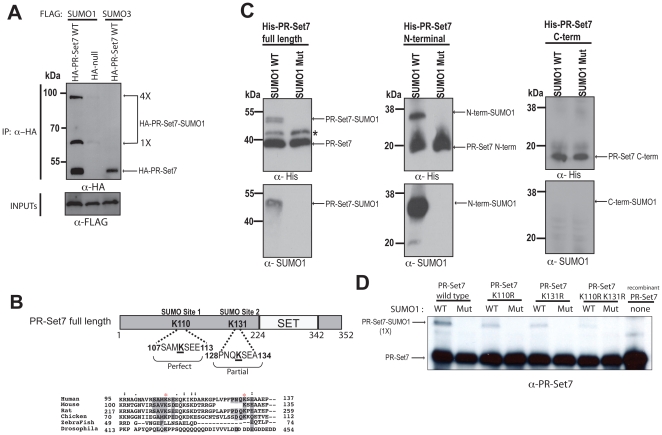
PR-Set7 is selectively modified by SUMO1 in cells and SUMOylated at K110 or K131 *in vitro*. (A) HEK 293 cells were co-transfected with the indicated expression plasmids. Cell extracts were prepared in the presence of NEM 2 days post-transfection, followed by immunoprecipitations using an anti-HA antibody. HA-bound samples were fractionated by SDS-PAGE prior to Western analysis using anti-FLAG or anti-HA antibodies. (B) Two putative PR-Set7 SUMOylation sites, one perfect and one partial, were identified using SUMOsp 2.0 [40]. ClustalX was used to align the sequence surrounding the putative SUMO sites of PR-Set7 in various organisms. Conserved residues are shaded and red asterisks mark K110 and K131. (C) Recombinant wild type PR-Set7, N-terminal (aa1–191) or C-terminal (aa192–352) fragments were used as substrates in *in vitro* SUMOylation assays. Reactions were fractionated by SDS-PAGE gel followed by Western analysis using anti-His and anti-SUMO1 antibodies. Asterisk (*) denotes an unknown contaminating protein. (D) Recombinant wild type PR-Set7, K110R, K131R or K110/131R double mutant were used as substrates in *in vitro* SUMOylation assays. Reactions were fractionated by SDS-PAGE followed by Western analysis using anti-PR-Set7 antibodies.

UBC9 functions as a SUMO E2 conjugating enzyme by directly binding and modifying target substrates [22], [23]. Due to its newly discovered interaction with PR-Set7, we hypothesized that PR-Set7 could be a potential target for SUMOylation. To determine if PR-Set7 could be covalently modified by SUMO *in vivo*, HEK 293 cells were co-transfected with an HA-tagged null or PR-Set7 plasmid and either FLAG-tagged SUMO1 or SUMO3 plasmids. Following N-ethylmalemide (NEM) treatment, a cysteine protease inhibitor used to preserve the SUMOylation of cellular proteins, lysates were collected and used for immunoprecipitations. Western analysis of the HA-bound material revealed two slow migrating bands in the HA-PR-Set7 sample containing FLAG-SUMO1 (Figure 4A). The estimated molecular weight of the bands suggests that PR-Set7 can be modified with either one (1×) or four (4×) SUMO1 moieties. In contrast, slow migrating bands were not detected in either the negative control (HA-null and FLAG-SUMO1) or FLAG-SUMO3 indicating that PR-Set7 is specifically modified with SUMO1 in these experiments.

### PR-Set7 is SUMOylated at K110 and K131 *in vitro*

UBC9 typically binds and modifies the consensus motif ΨKxD/E, where Ψ is a nonpolar amino acid [23]. Sequence analysis of PR-Set7 revealed two major putative SUMOylation sites in the N-terminal portion of PR-Set7 that could potentially bind UBC9 (Figure 4B). Both putative SUMO sites, K110 and K131, are conserved in PR-Set7 orthologues in higher vertebrates suggesting that modification of these residues could be biologically significant. Based on our findings we hypothesized that only the N-terminal portion of PR-Set7 would be covalently modified by SUMO1. To test this, recombinant full length PR-Set7 and the N-terminal_1–191_ and C-terminal_192–352_ fragments of PR-Set7 were used as substrates in *in vitro* SUMOylation reactions. Briefly, similar molar amounts of recombinant PR-Set7 proteins were incubated with either His-tagged wild type SUMO1 or a conjugation-deficient SUMO1 mutant in the presence of an E1 activating enzyme and the UBC9 E2 conjugating enzyme. Following incubation, the samples were fractionated by SDS-PAGE prior to Western analysis. In both the PR-Set7 full length and N-terminal_1–191_ samples, a slower migrating PR-Set7 band corresponding to one SUMO1 addition was consistently detected but was not observed in the C-terminal_191–352_ PR-Set7 sample (Figure 4C). Importantly, these bands were not detected in both the full length and N-terminal_1–191_ PR-Set7 samples when the SUMOylation-incompetent SUMO1 mutant was used in the reactions. These findings indicate that the PR-Set7 N-terminal_1–191_ fragment can be covalently modified with SUMO1 *in vitro*.

To determine if the two putative UBC9 consensus sites were required for SUMOylation of PR-Set7, both lysines were mutated to arginine (K110/131R) by site-directed mutagenesis. Recombinant wild type PR-Set7 and K110/131R mutant proteins purified from bacteria were used as substrates in the SUMOylation reactions described above. Western analysis of the SDS-PAGE fractionated samples revealed a slower migrating band corresponding to one SUMO1 addition to wild type PR-Set7 (Figure 4D). In contrast, this band was hardly detected in the K110/131R mutant suggesting that either K110 or K131 was the predominant SUMOylated site. To determine this, single lysine to arginine mutations were created for each site and recombinant proteins were purified for use in the SUMOylation assay described above. Unexpectedly, we found that both the K110R and K131R mutants could accept a single SUMO1 moiety (Figure 4D). These findings indicate that K110 or K131 of PR-Set7 are single SUMO1 acceptor sites and that SUMOylation of one of these sites precludes SUMOylation of the other site *in vitro*.

### The K110 and K131 SUMO sites are not required for nuclear localization of PR-Set7

**Figure 5:**
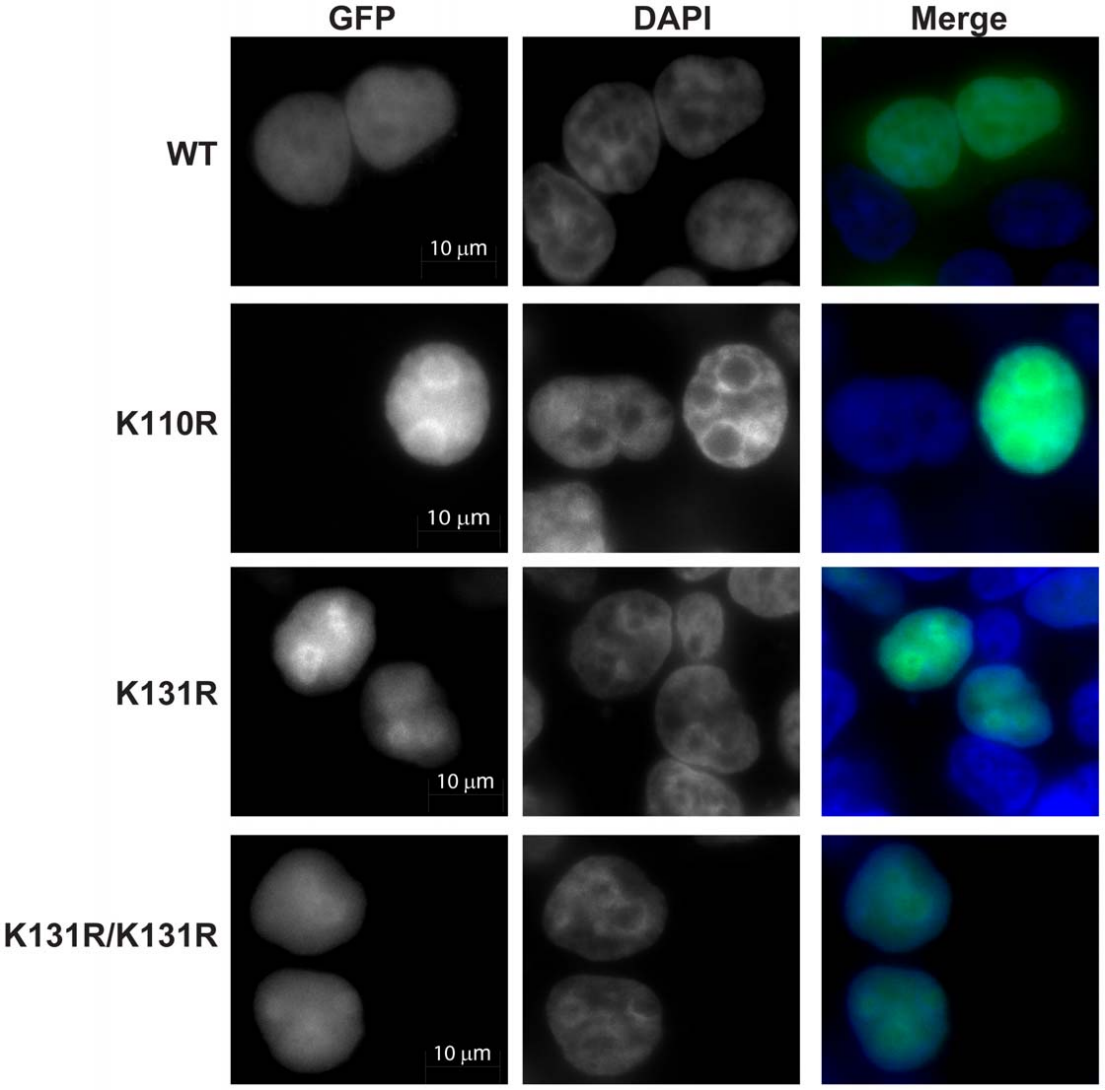
The K110 and K131 SUMOylation sites are dispensable for nuclear localization of PR-Set7. HEK 293 cells transfected with GFP-PR-Set7 wild type, K110R, K131R or K110R/K131R mutants (green) were fixed, stained with DAPI (blue) and visualized using a Zeiss Axio Imager upright fluorescence microscope.

Since previous studies demonstrated that SUMOylation of other chromatin-modifying proteins, such as the Aurora-B kinase and HDAC4 deacetylase, was required for their nuclear localization, we hypothesized that SUMOylation was also required for PR-Set7 nuclear localization [24], [25]. If this were the case, we reasoned that the PR-Set7 K110/131R mutants would display defects in nuclear accumulation compared to wild type PR-Set7. To test this hypothesis, a GFP-tagged null or wild type PR-Set7 were transfected into HEK 293 cells. Consistent with our previous findings, visualization of GFP by immunofluorescence microscopy confirmed that PR-Set7 was predominantly localized to the nucleus compared to GFP alone (Figure 5) [15]. Similar studies were performed with the GFP-tagged PR-Set7 mutants: K110R, K131R and K110/131R double mutant. Contrary to our hypothesis, all PR-Set7 mutants displayed nuclear accumulation similar to that of wild type PR-Set7 (Figure 5). These results indicate that PR-Set7 K110 and K131 are dispensable for targeting PR-Set7 to the nucleus.

### Depletion of PR-Set7 results in the increased expression of specific genes

**Figure 6:**
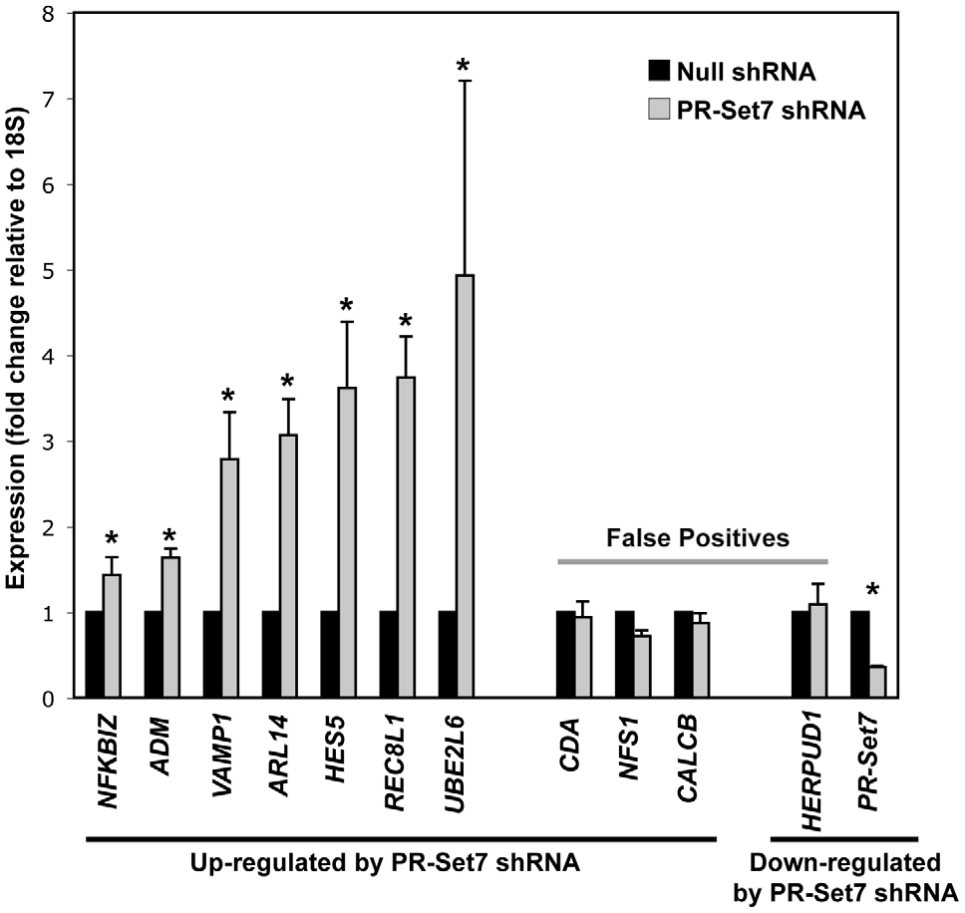
Depletion of PR-Set7 results in the derepression of specific human genes. Quantitative RT-PCR expression analysis of putative PR-Set7-regulated genes (Table 1) from control shRNA (black) or PR-Set7 shRNA (gray) transfected HeLa cells. Results are plotted relative to *18S* expression and normalized to control shRNA levels (y-axis). Error bars represent the standard error from three independent biological replicates. The student t-test was used to determine statistically significant changes at p<0.05(*). Seven of 10 identified genes significantly up-regulated in the absence of PR-Set7 by the expression arrays (Table 1) were validated whereas the only identified down-regulated gene was a false positive.

Although PR-Set7 and H4K20me1 were originally visualized in nuclear regions excluded of RNA polymerase II [5], [16], a recent genome-wide study in human CD4+ cells demonstrated that enrichment of H4K20me1 at the 5′ end of genes strongly correlates with highly expressed genes [17]. Therefore, it remained unclear if PR-Set7 and H4K20me1 function in gene activation or repressive pathways. To directly address this, we identified a panel of putative PR-Set7-regulated genes by first transfecting HeLa cells with a PR-Set7 shRNA plasmid or a null shRNA control plasmid. Whole cell lysates and total RNA were collected four days later to avoid potential indirect effects of the G2 cell cycle arrest observed at seven days following PR-Set7 shRNA transfection [7]. Depletion of PR-Set7 was confirmed by Western analysis in the PR-Set7 shRNA HeLa cells. Whole genome expression from two independent biological replicates of control and PR-Set7 shRNA samples was determined using Illumina BeadChip expression arrays. Computational comparison of the averaged expression of control versus experimental samples revealed 43 genes whose expression was significantly altered in cells lacking PR-Set7 (>2-fold, p<0.005) (Table 1). Of these, only 2 genes displayed decreased expression: *PR-Set7* and *HERPUD1*, which was found to be a false positive. The remaining 41 genes displayed increased expression. Quantitative RT-PCR of 10 of these genes confirmed 7 whose expression was significantly increased (p<0.05) in the absence of PR-Set7 (Figure 6). These findings strongly suggest that PR-Set7 functions predominantly in the repression of specific genes regardless of their basal transcriptional state, consistent with our previous reports [26], [27].

**Table 1:** Genes whose expression change >2-fold in cells lacking PR-Set7.

Genes Up-Regulated in the Absence of PR-Set7				
Gene Name	Probe ID	Avg. log2Δ(Null/PR-Set7 shRNA)	Avg. Fold Δ	p-value
CSAG3A	4010095	−3.415	10.666	0
CSAG2	6900377	−2.99	7.945	0
LOC653297	5670563	−2.773	6.835	0
LOC643425	5860504	−2.352	5.105	0
HES5	6590300	−2.127	4.368	0
UBE2L6	2070170	−2.103	4.296	1.00E-05
EPSTI1	5700725	−2.072	4.205	0
MAFA	5090241	−1.997	3.992	1.00E-05
PRIC285	5960343	−1.834	3.565	0
FLJ20035	7610053	−1.72	3.294	0
NFS1	7570411	−1.71	3.272	0.00166
REC8L1	70541	−1.675	3.193	0
CDA	5090372	−1.671	3.184	4.00E-05
FLJ11000	4670414	−1.533	2.894	0
MGC4677	6350189	−1.529	2.886	0.00109
LOC442578	3710711	−1.518	2.864	0.00021
CALCB	2230053	−1.478	2.786	7.00E-05
SAMD9	1240142	−1.432	2.698	1.00E-05
CD24	610437	−1.386	2.614	6.00E-05
CYP4X1	3290048	−1.276	2.422	1.00E-05
HERC6	6860482	−1.189	2.280	2.00E-04
LOC619383	6220112	−1.182	2.269	4.00E-05
ANKRD19	2480040	−1.163	2.239	0.00013
LOC440157	4280093	−1.158	2.231	0.00034
ARL14	3400209	−1.138	2.201	0.00182
FRAS1	270358	−1.131	2.190	0.0016
LOC132241	4200551	−1.126	2.183	0.00077
C21orf58	2070220	−1.122	2.176	5.00E-05
HOXC8	4640059	−1.098	2.141	7.00E-05
HS.154336	7040731	−1.07	2.099	0.00242
LOC642477	4570041	−1.063	2.089	0.00499
NRBP2	4490142	−1.043	2.061	1.00E-05
NFKBIZ	2470348	−1.037	2.052	0.00015
DHRS1	1470017	−1.031	2.043	0.00033
C14orf153	7160747	−1.03	2.042	0.0047
KIAA1641	4570075	−1.02	2.028	0.0012
VAMP1	6650639	−1.017	2.024	0.00103
HS.514745	4610041	−1.012	2.017	0
ADM	5670465	−1.01	2.014	0.00013
MCOLN2	5490068	−1.009	2.013	8.00E-05
EPDR1	6400044	−1.004	2.006	0.00022

Genes Down-Regulated in the Absence of PR-Set7				
Gene Name	Probe ID	Avg. log2Δ(Null/PR-Set7 shRNA)	Avg. Fold Δ	p-value
HERPUD1	4570458	1.186	0.440	7.00E-05
SETD8	2350735	1.938	0.261	0

### UBC9 facilitates PR-Set7-mediated gene repression

**Figure 7:**
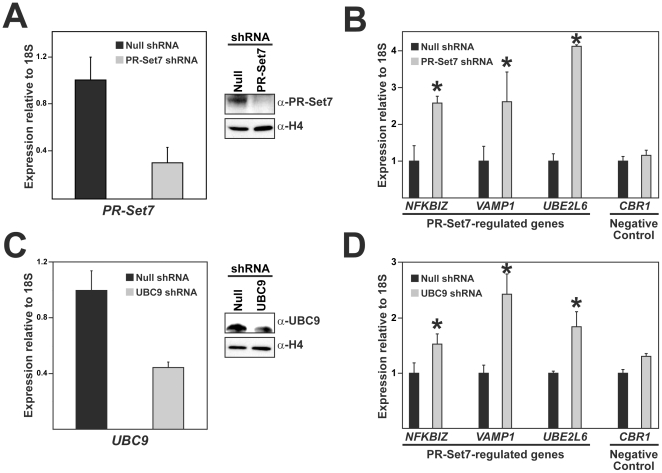
Reduction of UBC9 results in derepression of PR-Set7-regulated genes. HEK 293 cells were transfected with a null shRNA plasmid or shRNA plasmids designed to reduce PR-Set7 or UBC9 levels. Quantitative RT-PCR and Western analysis were performed to validate the reduced transcript and protein, respectively, of PR-Set7 (A) or UBC9 (C). Quantitative RT-PCR was performed to analyze the expression levels of three newly identified PR-Set7-regulated genes or the *CBR1* negative control gene in null shRNA (black bar), PR-Set7 shRNA (B) or UBC9 shRNA (D) cells (grey bar). Results were normalized to *18S* expression and plotted as the fold increase relative to null shRNA. Three technical replicates were used to generate standard deviations and statistical significance was calculated using the student-T test (asterisks are p≤0.05). Similar results were obtained in independent biological replicates.

Since both PR-Set7 and UBC9 were previously demonstrated to play a role in transcriptional repressive pathways, we hypothesized that PR-Set7 interaction with UBC9 was required for maintaining the transcriptional repression of genes regulated by PR-Set7 [26], [28]. The identification and confirmation of several PR-Set7-regulated genes made it possible to test this hypothesis directly (Figure 6). HEK 293 cells were transfected with the control shRNA plasmid or the PR-Set7 shRNA plasmid and samples were collected four days later. Both quantitative RT-PCR and Western analysis demonstrated that the PR-Set7 transcript and protein were nearly ablated in the PR-Set7 shRNA cells compared to null (Figure 7A). Quantitative expression analysis of three PR-Set7-regulated genes, *NFKBIZ*, *VAMP1*, and *UBE2L6*, confirmed that each was significantly increased in the absence of PR-Set7 (Figure 7B). In contrast, altered expression of the negative control gene, *CBR1*, was not observed [26]. The expression of the *18S rRNA* normalization control gene was consistent between all samples and the expression of other housekeeping genes were also unaltered in the PR-Set7 shRNA cells compared to control, as previously reported [26]. These findings indicate that PR-Set7 is required for the observed transcriptional repression of *NFKBIZ*, *VAMP1*, and *UBE2L6* in HEK 293 cells consistent with our findings in HeLa cells (Figure 6).

To determine if UBC9 was also required for the repression of the newly identified PR-Set7-regulated genes, HEK 293 cells were transfected with a null shRNA plasmid or a UBC9 shRNA plasmid that was previously reported to deplete cells of UBC9 [29]. Both quantitative RT-PCR and Western analysis confirmed a marked reduction of the UBC9 transcript and protein in the UBC9 shRNA cells compared to null (Figure 7C). Quantitative expression analysis of the PR-Set7-regulated genes, *NFKBIZ*, *VAMP1*, and *UBE2L6*, revealed that each was significantly derepressed in the UBC9 shRNA cells, although not to the same levels as observed in the PR-Set7 shRNA cells (Figure 7D). Importantly, altered expression of the *CBR1* negative control gene was not observed. Collectively, these findings indicate that UBC9 is required for the full repressive effects of PR-Set7-regulated genes.

## Discussion

In this study we sought to gain new insights into the role of PR-Set7 in transcriptional regulation by identifying and characterizing PR-Set7-interacting proteins. Using an unbiased yeast two-hybrid screen, we discovered that the UBC9 E2 SUMO conjugating enzyme directly binds PR-Set7 (Figure 1). Co-immunoprecipitation experiments confirmed that PR-Set7 and UBC9 interact in human cells although their binding appeared to be weak (Figure 2). It was previously shown that other UBC9-interacting proteins also display fairly weak interaction by co-immunoprecipitations [30], [31]. The addition of the protein cross-linking reagent, BMH, in these experiments greatly enhanced the detectable interaction between PR-Set7 and UBC9; even better than the p53 positive control. These findings indicate that PR-Set7 and UBC9 directly bind in human cells and strongly suggests that their interaction is transient.

The binding of UBC9 suggested that PR-Set7 is a target for SUMOylation *in vivo*. We found that PR-Set7 is preferentially modified with SUMO1 in human cells and that two evolutionarily conserved SUMO consensus sites adjacent to the catalytic SET domain, K110 and K131, were the primary sites of SUMOylation *in vitro* (Figure 4). Unexpectedly, our findings suggest that SUMOylation of one of these lysines precludes SUMOylation of the neighboring lysine. The significance of this observation remains unknown. It also remains unknown if K110 or K131 is the predominant SUMO1 acceptor site *in vivo* or if SUMOylation of each individual site functions separately in a distinct biological context. Further studies are required to resolve this. It is interesting to note that PR-Set7 accepts only a single SUMO1 moiety *in vitro* whereas the co-immunoprecipitation experiments demonstrate that PR-Set7 can accept one or four SUMO1 peptides. These results suggest that PR-Set7 may be SUMOylated at additional lysines other than K110 and K131 *in vivo*. Alternatively, a single PR-Set7 SUMO acceptor site may contain all four SUMO1 peptides as SUMO1 is capable of forming polymeric chains [32], [33]. If this is the case, it is likely that additional nuclear factors, such as an E3 ligase, are required for the polymeric SUMO1 modification of PR-Set7 *in vivo* since these components were not present in the *in vitro* assays. Additional investigation is necessary to resolve these possibilities.

Based on previous reports of other SUMOylated chromatin-modifying enzymes, we hypothesized that SUMOylation may affect PR-Set7 function by altering its ability to localize in the nucleus [24], [25]. Contrary to the hypothesis, we found that the absence of either or both SUMO acceptor sites, K110 and K131, did not result in any detectable changes in the cellular distribution of PR-Set7 (Figure 5). These findings indicate that K110 and K131 and their potential covalent modifications are dispensable for targeting PR-Set7 to the nucleus. However, we cannot definitively conclude that SUMOylation of PR-Set7 has no effect on its localization as there may be additional PR-Set7 SUMO acceptor sites that have yet to be identified.

It was recently reported that UBC9-dependent SUMOylation of the heterochromatin-associated proteins, Swi6 and Chp2, was required for transcriptional repression and the maintenance of heterochromatin stability in fission yeast [34]. Based on these findings, we hypothesized that UBC9 may function to similarly enhance the transcriptional repressive effects of PR-Set7. By employing expression arrays, we identified genes whose expression was significantly altered in the absence of PR-Set7 (Table 1). Our findings revealed that the vast majority of these genes displayed increased expression in cells lacking PR-Set7 strongly suggesting that PR-Set7 predominantly functions as a transcriptional repressor, consistent with our previous findings [16], [26], [27], [35]. Importantly, reduction of UBC9 by RNAi resulted in the significant derepression of a subset of genes regulated by PR-Set7 (Figure 7). The UBC9 RNAi cells did not achieve the levels of derepression observed in the PR-Set7 RNAi cells, however, this may be due to the inability to completely remove endogenous UBC9 by RNAi in these experiments. Regardless, our findings demonstrate that UBC9 is required to facilitate the full repressive effects of PR-Set7.

Since the main function of UBC9 is to conjugate SUMO moieties to target substrates, it is highly likely that the UBC9-mediated SUMOylation of PR-Set7 is required to promote the observed repression of PR-Set7-regulated genes, although this has yet to be demonstrated. How could the SUMOylation of PR-Set7 function in a transcriptional repression pathway? Recent findings demonstrated that UBC9-mediated SUMOylation of other chromatin-associated proteins, including p300, Elk-1 and reptin, was required for the recruitment of various histone deacetylases resulting in gene repression [36], [37], [38]. Therefore, one possible consequence of PR-Set7 SUMOylation may be to signal the recruitment of additional co-repressor proteins at PR-Set7 target genes to induce repression. Although our results strongly suggest that SUMOylation of PR-Set7 is dispensable for its nuclear localization, it is also possible that the addition of SUMO1 may be required to target PR-Set7 to chromatin and to specific genes. Lastly, it is possible that SUMOylation of PR-Set7 may alter its catalytic activity *in vivo* and, thereby, affect target gene expression since H4K20me1 is essential for gene repression [27]. These possibilities are not necessarily mutually exclusive and, therefore, additional investigation is required to determine the precise role of UBC9-mediated SUMOylation of PR-Set7 in transcriptional regulation.

## Materials and Methods

### Yeast-two-hybrid screening

Yeast-two-hybrid screen was performed using the MATCHMAKER GAL4 Two-Hybrid System 3 protocol (Clontech). The bait plasmid was created by inserting PCR-amplified fragment encoding full length human PR-Set7 (Gene ID: 387893) into the *Nde*I-*Bam*HI restriction sites of pGBKT7 plasmid. The GAL4-DBD-PR-Set7 plasmid or pGBKT7-null were transformed into AH109 yeast strain and each was mated to pre-transformed human *HeLa* Matchmaker cDNA library in the Y187 yeast strain. Mated yeast were selected on synthetic medium without tryptophan and leucine (DDO) and histidine (TDO) and adenine with X-alpha-galactosidase (QDO+X-α-Gal) to screen for *ADE2*, *HIS3* and *MEL1* expression. TDO and QDO surviving colonies were re-streaked on corresponding selective plates to assure maintenance of the correct phenotype followed by plasmid isolation using YEASTMAKER Yeast Plasmid Isolation Kit (Clontech) and DNA sequencing. Candidate clones were co-transformed with the PR-Set7 bait plasmid into the AH109 yeast strain and grown on selective media to confirm the interaction.

### Immunoprecipitations

The AH109 yeast strains were grown at 30°C in 100 mL of DDO medium to OD_600_∼1.0. Yeast extracts were prepared by glass-bead lysis in immunoprecipitation (IP) buffer (50 mM Tris-HCl, pH 7.5, 50 mM NaCl, 1 mM PMSF, 10 µg/mL Pepstatin A, 10 µg/mL Leupeptin/Aprotinin and 1% Triton-X-100). Reactions containing 0.5 mg of total protein and 50 µL of a 50% EZview Red anti-HA affinity gel slurry (Sigma) in a 1 mL final volume of IP buffer were incubated over night at 4°C. Beads were washed three times with 1 mL of wash buffer (50 mM Tris-HCl, pH 7.5, 100 mM NaCl, 1 mM PMSF, 10 µg/mL Pepstatin A, 10 µg/mL Leupeptin/Aprotinin and 1% Triton-X-100). The bound material was eluted by boiling in SDS loading buffer and fractionated by SDS-PAGE. HEK 293 cells were transfected using Lipofectamine 2000 according to the manufacturer's instructions (Invitrogen). Cells were incubated in DMEM medium with or without 10 µM BMH for 10 min at room temperature 24 hours post-transfection. Whole cell IP lysates were made by resuspending 2×10^7^ cells in 500 µL of IP buffer (50 mM Tris-HCl pH 7.0, 150 mM NaCl, 0.5 mM DTT, 1% NP-40, 1 mM PMSF, 1 µg/mL pepstatin and 1 µg/mL aprotinin/leupeptin), followed by an overnight 4°C incubation with 40 µL of pre-equilibrated 50% EZview Red anti-HA or anti-FLAG affinity gel slurry (Sigma). Beads were washed extensively with IP buffer prior to eluting bound material by boiling in SDS loading dye. Samples then were fractionated by SDS-PAGE prior to Western analysis.

### SUMOylation and binding assays

The *in vitro* SUMOylation assay was performed with a SUMOlink Kit according to the manufacturer's instructions (Active Motif) using purified recombinant proteins as substrates. Glutathione S-transferase (GST)-null, GST-UBC9 and His-S-tag-PR-Set7 recombinant proteins were induced in BL21 *E. coli* and purified by either glutathione-conjugated Sepharose 4B beads or Ni-Sepharose High Performance agarose beads (GE Healthcare), respectively. Recombinant His-S-tag-PR-Set7 (10 µg), 3 µg of anti-S-tag antibody and 20 µL of pre-equilibrated Protein-A beads in a 300 µL final volume in IP buffer were incubated overnight at 4°C. Purified GST-UBC9 (5 µg) or GST alone (3 µg) were added to the reaction and incubated at 4°C for 8 hours. For GST pull-downs, 3 µg of GST-UBC9 or GST alone were incubated with 15 µL of *in vitro* translated [^35^S]-labeled His-PR-Set7 proteins (Promega) in IP buffer overnight at 4°C followed by a 4 hour incubation at 4°C with 20 µL of pre-equilibrated glutathione-conjugated Sepharose 4B beads (GE Healthcare). Beads were washed with IP buffer and bound proteins were eluted in SDS loading dye prior to fractionation by SDS-PAGE and Western analysis.

### Western analysis

Western analysis was performed as previously described [16] using the following antibodies and dilutions: PR-Set7 (1∶1 k; Cell Signaling), UBC9 (1∶500; Santa Cruz Biotechnology), FLAG (1∶2 k; Sigma), His (1∶1 k; Novagen), GST (1∶5 k; Upstate), SUMO1 (1∶4 k; Active Motif).

### Immunofluorescence

HEK 293 cells were transfected with wild type or mutant GFP-PR-Set7 plasmids using BioT (Bioland Scientific). Cells were fixed 24 hours post-transfection and counterstained with DAPI as previously described [39]. Staining was visualized using a 63× objective on Zeiss Axio Imager upright fluorescence microscope with ApoTome and images were analyzed using Adobe PhotoShop CS5.

### Gene expression analysis

Control, PR-Set7 or UBC9 shRNA plasmids were transfected into HEK 293 using Lipofectamine 2000 (Invitrogen) as previously described [27], [29]. Total RNA was isolated (Qiagen) 4 days post-transfection for UBC9 shRNA or PR-Set7 shRNA cells and cDNA was created using a Reverse Transcriptase Kit (Applied Biosystems). Quantitative real-time PCR was performed in triplicate using 10 ng cDNA. Fold change was calculated using 2∧(−ΔΔCt) and plotted relative to control shRNA cells. Primers: *NFKBIZ* (5′-TGGTTGATACCATTAAGTGCCTA, 3′-GTAAGCCTTTGCATTCACAAAA), *VAMP1* (5′-AGCATCACAATTTGAGAGCAGT, 3′-GTGTTGAGAGAGCAAACAGAGG), *UBE2L6* (5′-CCATGATCAAATTCACAACCA, 3′-TTCGGCATTCTTTCTGAACA), *CBR1* (5′-TGATCCCACACCCTTTCATA, 3′-AGCTTTTAAGGGCTCTGACG), *18S* (5′-AACTTTCGATGGTAGTCGCCG, 3′-CCTTGGATGTGGTAGCCGTTT), *ADM* (5′-GGACGTCTGAGACTTTCTCCTT, 3′-ACGACTCAGAGCCCACTTATTC), *ARL14* (5′-AGAACTGTTTGGGGCTGTTACT, 3′-CACTGCAAAGCTTCTTCACTTT), *HES5* (5′-AGCTACCTGAAGCACAGCAA, 3′-GAAGTGGTACAGCAGCTTCATC), *REC8L1* (5′- AATTCCAGGAACAACTGCAAA, 3′-TTGGAACTTCAATCTTTCTCCTT), *CDA* (5′-CAAGATGATTTTATCTCTCCATGTG, 3′-GACATCTTTATGAAGTTCTCCAGGT), *NFS1* (5′-TCTATATGGATGTGCAAGCTACAA, 3′-TTGCTATGTTGTTGGATTCAGTAG), *CALCB* (5′-CAGATGAATGACTCCAGGAAGA, 3′-CTGTGATTCTGGCTTCTGGTAG), *HERPUD1* (5′-TAAATCGAGATTGGTTGGATTG, 3′-GTTATTGTTGGGGTCCTGATTT).

### Whole genome expression analysis

HeLa cells transfected with control or PR-Set7 shRNA plasmids were selected with 5 µg/ml puromycin before total RNA isolation (Qiagen) 4 days following transfection. Two independent biological replicates of each were hybridized to the Illumina Human WG-6 v.3.0 Expression BeadChips (GEO accession: GSE30361). The raw intensity values of the probes were processed using the Bead Studio application. To efficiently analyze the data, the bead-summary files were produced and subsequently processed using the *beadarray* library of the BioConductor framework in the R programming environment. Probes that had a log_2_ difference between PR-Set7 shRNA and control samples with an absolute value equal to or greater than 1 at a statistically significant level (p<0.005) were selected for further analysis. Genes whose expression are known to be significantly altered by indirect effects associated with RNA interference, such as interferon or mitogen-induced genes, were excluded from the analysis.
